# The multidisciplinary team meeting in the UK from the patients’ perspective: comments and observations from cholangiocarcinoma patients and their families

**DOI:** 10.2147/IJGM.S145029

**Published:** 2017-09-18

**Authors:** Helen Morement, Rachel Harrison, Simon D Taylor-Robinson

**Affiliations:** 1 AMMF – The Cholangiocarcinoma Charity, Enterprise House, Stansted, Essex; 2 Department of South East Asia, School of Oriental and African Studies, London; 3 Division of Digestive Health, Department of Surgery and Cancer, Imperial College London, London, UK

**Keywords:** cholangiocarcinoma, multidisciplinary team meeting, management, patient perspectives

## Abstract

**Background:**

The multidisciplinary team (MDT) meeting has become the hallmark for cancer care in the UK. While standardizing care through adherence to guidelines, the MDT process can make the decision-making process somewhat remote from the patient perspective. The Cholangiocarcinoma Charity (AMMF) is the UK’s only cholangiocarcinoma charity and is at the forefront of patient empowerment for those with this condition and for their families. It provides much needed support not only via personal contact but also through its website and on the social media platforms, Facebook and Twitter.

**Methods:**

AMMF conducted a survey of patient attitudes to and experience of the MDT process through a simple questionnaire posted on Facebook in 2014. We report the results of the responses received, which we believe are worthy of further thought.

**Findings:**

In the main, while treatment decisions are not queried, there is distress at the lack of involvement, the lack of representation, the lack of communication and at not knowing who to approach for answers to questions.

**Conclusion:**

This snapshot, although small, provides some insight to clinicians not to forget the constituency they serve, as communication is all important.

## Introduction

The multidisciplinary team (MDT) meeting has become a familiar and mandatory part of the care pathway for patients with cancer or suspected cancer in the modern age.^1^ There has been much written about the efficiency of thought from the professional perspective with the opportunity for physicians, oncologists, radiotherapists, surgeons, palliative care physicians, radiologists and histopathologists to be able to meet on a weekly basis to guide and monitor a joint approach to the care of individual patients within a hospital trust or network of hospitals at a local or regional level.^2^ Furthermore, much has been written about the health economics of such an approach to the point that it seems the supremacy of the MDT or its wisdom is no longer questioned.^3^,^4^ While a well-functioning MDT is undoubtedly a benchmark for good quality care, in reality,^5^–^7^ many MDTs in the UK are hard pressed with a tsunami of work to get through and little time to discuss all cases properly in the hour or so allotted, often at the beginning of the day, before “real work” begins. In addition, the hard fact is that while each patient discussed should have a clinical advocate (who knows them) present at the MDT, often this is not the case in the UK with decisions made by a well-meaning team who are usually bound by guidelines and who make decisions, based on radiological and histological evidence, in the absence of direct patient contact. As this process is driven by a panel of experts, often quite remote from the patients and their families, it has the potential to give rise to weaknesses in approach to patient communication, one which has the potential not only to cause emotional distress and anxiety to those undergoing diagnosis and treatment, but which threatens to exclude the patient as a “conscious” and involved participant in their own care and recovery.

In the spirit of appraisal, it is worth considering what patients think about the MDT process and what it means to them. Cholangiocarcinoma or bile duct cancer is becoming steadily more common, and it is a particularly cruel disease in that it presents rapidly and usually too late for meaningful intervention, giving patients and their families little time to adjust to a condition, where incidence and mortality are almost the same.^8^ The Cholangiocarcinoma Charity (AMMF) is the UK’s only dedicated cholangiocarcinoma charity, and it supports patients and their families coming to terms with difficult diagnoses, in addition to supporting medical research on cholangiocarcinoma (www.ammf.org.uk).

The charity is active on the social media platforms, Face-book and Twitter, to keep its followers informed of clinical and research developments in the field. As Chairman of AMMF, Helen Morement was approached to speak on patient attitudes toward the MDT and their experiences at a national forum in Liverpool, UK. Before doing so, she commissioned a questionnaire using the AMMF Facebook page to ascertain the views of patients and their families regarding the MDT process for cholangiocarcinoma. We report the results of the responses to this Facebook questionnaire.

Although this descriptive approach is unusual in that we report the results of a social media survey where the respondents are self-selected, and it therefore lacks scientific validity in terms of standardization and statistical methodology. The patient responses are reported without subjective editing, and in the spirit of 360 degree feedback, they are worthy of further thought.

## Methods

Prior to speaking on the subject, “MDT – The Patients’ Perspective” at the European Society of Surgical Oncology (ESSO) and the British Association for Surgical Oncology (BASO) MDT Course on November 1, 2014, the AMMF’s Chairman Helen Morement used the charity’s Facebook page to invite supporters to supply answers, comments and thoughts, either openly on the public site or privately by email on the following series of questions:

1. If you, or someone you are close to, has been diagnosed with cholangiocarcinoma, were you/they told that your/their treatment would be discussed at an MDT meeting?
2. If you were told about an MDT meeting, did you know what this meant and did you understand what would be happening?
3. Did you have an opportunity to ask questions about this, and were they fully answered to your satisfaction?
4. How were the decisions on treatment options reported back to you?
5. Did you have an opportunity to ask questions about this?

As this was an entirely empirical exchange on a busy social media site, run by the lay public for the lay public, and aimed at promoting patient welfare, research ethics to carry out the questionnaire was considered unnecessary by the AMMF Charity Board of Trustees. We present completely anonymized data from the online survey. However, the majority of respondents, whether commenting openly on Facebook or via email, agreed to their responses and to their names being shared publicly.

All participants included in this report were emailed and gave their subsequent permission for the publication of the collective, anonymized findings for educational, research and health care quality improvement purposes. Their information provided the detail for the oral presentation in Liverpool, and the responses are shown in full in the “Results” section.

The fully anonymized responses to the questionnaire document were circulated as a printed handout to all the attendees at the ESSO-BASO Cancer MDT Course in November 2014 with the respondents’ prior agreement.

## Results

The following are the unedited, empirical responses to the questionnaire posted on the AMMF Facebook page:

### Respondent 1

We were told on the day that the diagnosis was given to us that my father would be discussed by an MDT at the regional teaching hospital and that numerous professionals attended these meetings.

The clinical nurse at district general hospital said she had rushed through our appointment at which the doctor told us it was cancer, so my father’s case could be discussed at that Wednesday’s meeting, otherwise we would have had to wait until the following Wednesday for his case to be discussed. We were not told we could ask questions. We did not get much feedback apart from being told that my dad was not able to be operated on, but that chemotherapy was an option to control, not to cure it. I suppose I would have liked the chance to speak to some of the people at the meeting and maybe ask further questions, but at least we were told this much.

Of course I completely understand that we are not allowed to attend the meetings, nothing would ever get decided. I agree that more could be done to make us feel fully involved. However, I appreciate that with the number of cancer patients ever increasing then there has to be a limit to that involvement.

I suppose I am trying to understand from the medical profession’s point of view as well, but that is not my problem to worry about when I have enough to deal with, and I have never felt I could just pick up the phone or email someone about this, because no one has told us this is something we can do. If we had access to a patient advocate that could make this whole traumatic experience a little easier.

### Respondent 2

I recall being told after diagnosis that my case was to be discussed at a meeting in another hospital and that this was attended by a range of medical specialists from a wide area.

MDTs exist in other professional areas and I had attended many over the years. Some of these events had been very positive and some less so, dependent on those attending and particularly the “chair or lead professional”. Decisions taken by committee can sometimes involve compromise. I do remember thinking I hope the key individuals are all actually there. I also remember that this was an unusual approach and that this thickened the fog of first, a cancer diagnosis and second, one that was complex.

The effect on the patient of the apparent delay in agreeing a way forward, even for a day or a weekend, should never be underestimated. This sends a very mixed message. The lack of the usual pathway of one individual physician or surgeon explaining what procedure they intended to carry out was unsettling. Would literature on what the MDT constitutes be of help? I am not sure for me, but for those around me, I think it would.

### Respondent 3

We have been told on numerous occasions about my husband’s case being discussed at the MDT meetings, which have mainly been reported back to us via the oncologist either by telephone or at our next clinic appointment, which caused inevitable delay.

### Respondent 4

Having to wait for an MDT meeting can be frustrating, but it is necessary to have all the specialists together at once. As a nurse, I fully understood what the MDT was and what would be discussed, but it was frustrating that I had to constantly explain the medical terminology to my mother and the rest of the family. I often felt medical and nursing staff spoke to me rather than my mother, who was the patient.

The MDT decisions on treatment options were discussed and we were given time to ask questions. In fact, the doctor explained things clearly and used appropriate sketches to explain why and allowed my mother to take them home. When my mother had her chemotherapy regime explained, it was also written down for her. My mother’s case was discussed in numerous MDTs and we were always aware when these were taking place and why. The outcomes were discussed and on more than one occasion the doctor also rang me (at my mother’s request) to inform me.

### Respondent 5

My mother had her case dealt with by an MDT and although we were grateful that such expertise was “on the team”, I recall feeling immense frustration that their weekly or even fortnightly meetings were so rigidly timed. For example, there could be an MDT meeting on a Wednesday afternoon, but if my mother had a scan on a Thursday morning she could have to wait up to 2 weeks for the results to be discussed.

I recall several calls to the hospital begging staff to run scan results down to the appropriate secretary, so that they could be included in that afternoon’s discussion. For all cancers, and specifically for aggressive, time-critical cancers like cholangiocarcinoma, I wondered why there was not a better solution to this. Two weeks could mean all the difference. Technology has surely evolved?

If I were going to add another point about the MDT, it would be nice to understand how and why decisions had been reached and to receive feedback.

### Respondent 6

At the time of my husband’s first operation, there was no mention of MDT, because it was originally thought he had pancreatitis but, 2 weeks later we got the cholangiocarcinoma diagnosis as a result of the histology report. We were then told that there would be an MDT meeting the next Tuesday, a weekly event, involving, among others, the liver surgeon, who had performed the operation, and the oncologist and the next steps would be discussed. As my husband was still in the high dependency unit (HDU) due to complications from post-operative methicillin-resistant *Staphylococcus aureus* (MRSA), the outcome of the meeting was discussed with us in the ward with plenty of chances to ask questions.

A year later when the cancer had spread to the duodenum, it was very different. There was an MDT meeting, but he was in a different ward and we had great difficulty getting any information. Indeed, the impression we got was that the professionals could not agree on next steps. Perhaps, the decision was more difficult to make, I do not know, but it was upsetting as we felt we were being kept in the dark.

My husband would never make a fuss, but I did the “fuss bit” for both of us, desperate for information. Two extreme opposite experiences, and I know which we preferred. It was so much better first time round to feel involved in the post-MDT deliberations and have the opportunity to ask questions

### Respondent 7

My parents and I were told by our local district general hospital that the regional teaching hospital team had discussed and diagnosed my father’s cancer at an MDT. They then transferred my father to the regional teaching hospital for his stent procedure. Our local district general hospital had told us my father had a few months to live, but the regional teaching hospital offered chemotherapy and disagreed with the prognosis. We felt that there were mixed messages coming from the MDT.

Six months later, my father’s case was discussed again at an MDT and it was decided nothing could be done. This news was broken to my father by the Macmillan nurse and he never saw the consultant again. Both times we were told after the MDTs and did not have a chance to put our questions forward. I often had to try to telephone or even email them to ask questions and get answers.

### Respondent 8

I knew what an MDT was, but it has never been mentioned to me. My first experience was in France, while my second experience was at a district general hospital in the UK. Once it had been discovered that I had tumor recurrence at the MDT review, my oncologist simply told me that if I thought I had a tough time last time, it was nothing compared to what will happen. She told me I had 18–21 months to live (this was in July 2013), then told me to have a cup of coffee before driving the 45-minute journey home. I was on my own. There was no discussion at all regarding any treatment.

My third experience was at a regional teaching hospital. The oncologist there was excellent at explaining what and when things were going to happen. He did not mention an MDT specifically, but I certainly got the impression that my case had been discussed with others. He explained that I was to have chemotherapy and referred me to another teaching hospital for treatment.

My fourth experience was at the next teaching hospital. My oncologist explained every part of my treatment and, upon asking, explained why radiotherapy and surgery were not options in my case. I was happy with the decisions made and again felt my case had been discussed although there was never a mention of an MDT.

My only comment is that although I appreciate it would not be appropriate for a patient to attend an MDT, I think it would be helpful for members of the MDT to meet the patient for them to assess strength of character, and attitude, which could have an impact on decisions made. It could also be of benefit to the patient to gain confidence in the decision-making process.

### Respondent 9

I can honestly say I was told nothing. I went to see a liver consultant at the hospital. I was taken into a room full of people. I had no idea who they were. A consultant introduced himself and told me I needed a major operation as I had a tumor on my liver that needed to be removed. He said you have us baffled – there is no cancer showing in your body, but you need a major operation. MDT was never mentioned. I can recall being so confused that I cancelled the operation because of lack of knowledge. It was only when I was appointed a new consultant that I was invited to his clinic to ask questions and fully discuss my options and be told exactly what to expect.

### Respondent 10

We were told very early on that my sister’s case would be discussed with an MDT. I am not sure that the word “MDT” was used but she knew that every Friday afternoon a meeting was held with surgeons, oncologists, etc. As a nurse, I knew what an MDT was, so I could explain it.

Whenever a decision was being made at the MDT, my sister knew she was being discussed at the meeting and was told by her surgeon that he would ring her after. This was always after 5 pm on a Friday afternoon. He always phoned and I have to say the news she was given was always extremely hard to hear. It had to be done by phone, because of the distance involved and the rapidity of decisions being made and plans put in place. She was always allowed to ask questions but often more cropped up after the phone call and she had to wait until Monday to contact a professional. That is no-one’s fault and not a criticism but maybe a Friday 3 pm MDT meeting is not the ideal time if you are delivering difficult news.

### Respondent 11

I had an MDT before my surgery and took the advice that any delay would cause further problems down the recovery road. I had a diagnosis to operation (Whipple’s procedure) in two weeks. I believe that saved my life.

## Discussion

This small snapshot of responses made on social media and by email, giving the experiences and thoughts of cholangiocarcinoma patients and their families following the Facebook questionnaire conducted by AMMF on the perception of the MDT, is worthy of further reflection, although we acknowledge that the survey was not designed to be representative of all experience and is entirely subjective and nonscientific. Although, in general, treatment decisions are not queried, there is respondent distress at the lack of involvement, the lack of representation, the lack of communication, and at not knowing who to approach for answers to questions.

While our methodologies may be criticized for being unrepresentative, the concerns raised by the patient respondents have been recurring themes at national public information fora organized by the AMMF over the past couple of years across the UK.

There does genuinely seem to be confusion among cholangiocarcinoma patients and their families over the decision-making process used at MDT meetings and anxiety over whether anyone is present who might know the patient and act as their advocate, particularly if the MDT meeting is taking place at a different hospital from where care is being given. However, the two overwhelming issues seem to be: first, the perception that the MDT process may lengthen the time to a decision on the patient management pathway, particularly if the MDT convenes less frequently than every week and second, that communication between the MDT and the patient is often poor, with the MDT being seen as a remote body who sit in mysterious, secret conclaves, a bit like Catholic Cardinals electing a new Pope! With the emphasis on streamlining procedures and improving cost-effectiveness of cancer care, the MDT meeting has been championed as the guardian of quality and the champion of standardization of care according to national guidelines.^4^ It should also be borne in mind from the health care management point of view that in the context of cholangiocarcinoma and many other cancers, diagnosis is often difficult and the MDT framework provides the clinician with the framework for a consensus on diagnosis. However, it is certainly the case that depersonalization can set in with patients with their lives at stake, being treated as numbered cases who are dealt with quickly and often dispassionately. Furthermore, if the responsible consultant is not present at the meeting, the communication of decisions can be delayed and outcomes can be conveyed in less than ideal circumstances.

## Conclusion

This social media survey led by the lay public for the lay public is nonscientific in its methodology and serves only to alert the medical profession to potential problems in the MDT process. Furthermore, what has been reported for cholangiocarcinoma, a rare cancer in the UK, cannot be extrapolated to all MDTs for every cancer, where more favorable patient experiences have been reported. 9–12

Nevertheless, in an era of patient-centered care, we would suggest that clinicians who have personal knowledge of their patient, their character and attitude, always attend MDTs to act as their advocates, as very often decisions can be made on following rigid guidelines without knowledge of biological fitness or patient wishes. We would also suggest that in an age where efficiency is the aesthetic goal, but where there is an ever busier clinical load, time is made to impart decisions with compassion and an opportunity to ask questions. While this is done well in most cases, what seem to be basic human rights to the lay public are sometimes forgotten in the rush to see the next patient.

While this may be understandable in terms of the pressures of workload on medical professionals, it is also essential to consider the subjectivity of the patient as a vital element of the treatment process. This is not only from the perspective of basic human rights, agency and dignity but also in relation to the very practical concerns of active patient participation in their own health care agenda and the benefits of this participation to all those concerned. An alternative but perhaps more radical approach to patient participation is to have patients and their families present at MDT meetings.^9^ The possibility of patients taking part in the MDT is controversial, but it is something that many would wish for in a more patient-centered health care system. Such approaches need to be discussed, but are not without the bounds of future possibility.
